# Unilateral Hemispheric Hyperperfusion in Intravascular Large B-cell Lymphoma

**DOI:** 10.7759/cureus.63417

**Published:** 2024-06-28

**Authors:** Daichi Imamura, Satoru Fujiwara, Hiroki Amagase, Michi Kawamoto

**Affiliations:** 1 Department of Neurology, Kobe City Medical Center General Hospital, Kobe, JPN; 2 Department of Hematology, Kobe City Medical Center General Hospital, Kobe, JPN

**Keywords:** hyperperfusion, intravascular large b-cell lymphoma, intravascular lymphoma, epilepsy, arterial spin labeling (asl)

## Abstract

The diagnosis of intravascular large B-cell lymphoma (IVLBCL) is often challenging owing to its nonspecific clinical manifestations and imaging findings. Herein, we present a rare case of IVLBCL in which seizure was the initial symptom, and unilateral hemispheric hyperperfusion on arterial spin labeling (ASL) was the only abnormal finding observed on brain magnetic resonance imaging (MRI). A 68-year-old male with a history of hypertension and type 2 diabetes was transferred to the emergency room owing to the sudden onset of altered consciousness and abnormal behavior. Upon arrival, the patient was disoriented and confused, and cerebrospinal fluid analysis revealed pleocytosis and elevated protein level. Even after the administration of acyclovir and antiepileptic drugs, his consciousness remained impaired, with repeated transient right hemiparesis indicating a focal seizure. The initial and follow-up MRI scans showed no obvious abnormalities in diffusion-weighted imaging (DWI), T2-weighted imaging, or susceptibility-weighted imaging (SWI); however, ASL revealed markedly increased blood flow to the left hemisphere. Subsequently, the rapid elevation of serum lactate dehydrogenase (LDH) and soluble interleukin-2 receptor (sIL-2R) levels after admission led to the diagnosis of IVLBCL by random skin biopsy and bone marrow examination. Despite the initiation of chemotherapy, the patient developed tumor lysis syndrome and succumbed to multiple organ failure. This case underscores the importance of considering IVLBCL in adult patients with refractory seizures and highlights the potential utility of ASL on MRI for early diagnosis.

## Introduction

Intravascular lymphoma (IVL) is a rare form of lymphoma characterized by the infiltration of tumor cells within the lumina of small vessels, particularly the capillaries and postcapillary venules. The diagnosis of IVL is often difficult because of the variety of clinical manifestations secondary to this pathological mechanism that can occur in any organ. Central nervous system (CNS) involvement is reported relatively frequently with 75%-85% [1]; however, there are usually no pathognomonic signs or symptoms. Moreover, magnetic resonance imaging (MRI) findings are also usually inconclusive, except for some categories of signs, such as the nonspecific hyperintensity of white matter lesions suggestive of small-vessel ischemic disease, which have been reported [2]. The diverse clinical presentations often pose diagnostic challenges, with many cases historically diagnosed only postmortem. Herein, we report the case of a patient with intravascular large B-cell lymphoma (IVLBCL) who characteristically presented with seizures as an initial symptom and impressive MRI findings without typical findings.

## Case presentation

A 68-year-old right-handed male with a history of hypertension and type 2 diabetes was transferred to the emergency room owing to the sudden onset of altered consciousness and abnormal behavior that occurred while at work.

**Figure 1:**
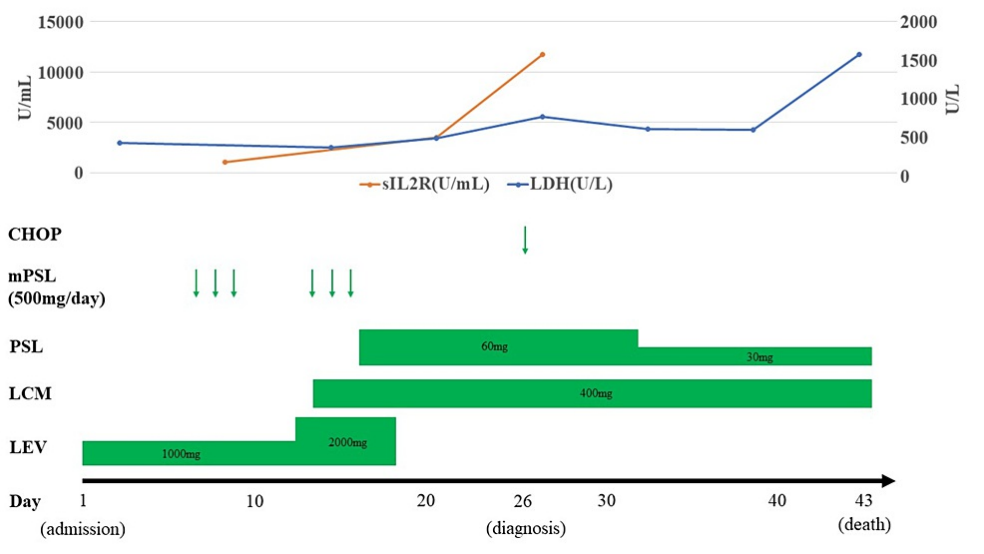
Clinical course (A) Laboratory data, (B) treatment LDH: Lactate dehydrogenase; sIL-2R: soluble interleukin-2 receptor; mPSL: methylprednisolone; PSL: prednisolone; LCM: lacosamide; LEV: levetiracetam; CHOP: cyclophosphamide, hydroxydaunorubicin, vincristine, and prednisolone

Upon arrival, his vital signs were as follows: body temperature, 36.6°C; blood pressure, 146/86 mmHg; heart rate, 118 /min; respiratory rate, 24 /min; and oxygen saturation, 96% (room air). The patient was disoriented and confused with a Glasgow Coma Scale of E4V3M5. This required sedation each time for imaging tests or lumbar puncture. Neurological examination revealed conjugate deviation to the left, unresponsiveness to commands, and perseveration, but no obvious limb paralysis and sensory disturbances were observed. Laboratory tests revealed moderately elevated serum lactate dehydrogenase (LDH) (417 U/L) and soluble interleukin-2 receptor (sIL-2R) (1,081 U/ml) levels without electrolyte, blood cell abnormalities, and hypoglycemia (Figure 1A). Cerebrospinal fluid analysis on the admission day revealed pleocytosis (15 /μL, 100% mononuclear) and elevated protein level (319 mg/dL) without the cytological evidence of atypia. Electroencephalography (EEG) revealed no epileptic discharges; however, intermittent, irregular delta activities were scattered in the left hemisphere. On the first MRI scan after admission, no abnormalities were observed on diffusion-weighted imaging (DWI) or T2-weighted imaging.

**Figure 2:**
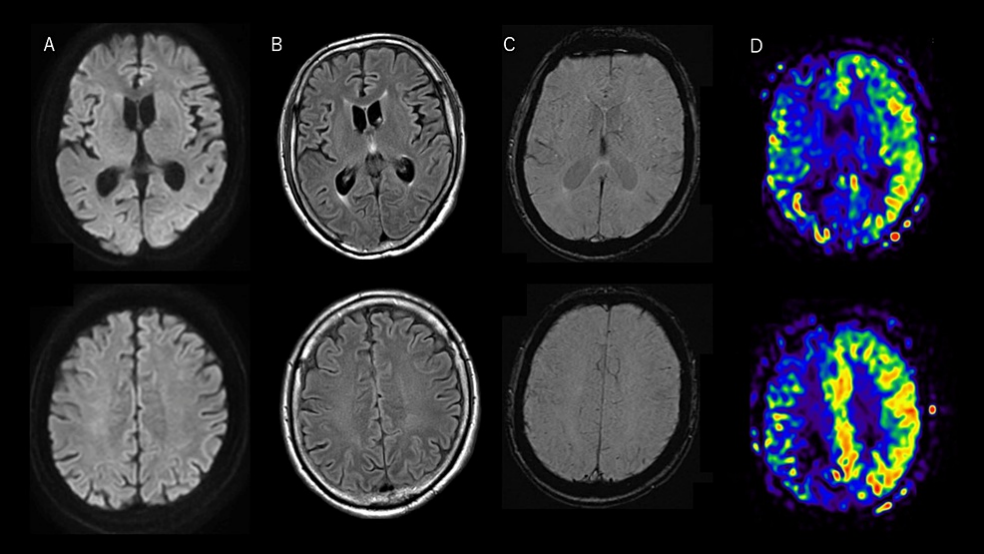
Magnetic resonance imaging after admission (A) Diffusion-weighted imaging (DWI), (B) fluid-attenuated inversion recovery (FLAIR), (C) susceptibility-weighted imaging (SWI), and (D) pulsed continuous arterial spin labeling (pCASL) with post-labeling delay (PLD) of 1,500 ms DWI/FLAIR/SWI did not reveal any abnormalities. However, ASL detected unilateral hemispheric hyperperfusion

Considering the acute symptomatic seizures associated with meningoencephalitis, we initiated the administration of acyclovir and levetiracetam (1,000 mg/day) (Figure 1B). After hospitalization, his conjugate deviation to the left resolved within a few days, and his level of consciousness partially improved; however, he continued to show a tendency toward verbosity and attention deficits. In addition, on the 6th and 13th days of admission, he repeatedly developed transient right hemiparalysis lasting for approximately five minutes, indicative of a focal seizure with no full recovery of consciousness. EEG at the next day of transient hemiparalysis also showed intermittent irregular delta activities in the left hemisphere and no epileptic discharges. We increased the dose of levetiracetam to 2,000 mg/day and added lacosamide (400 mg/day), followed by steroid pulse therapy (methylprednisolone 500 mg daily for three days twice), but there was no improvement in the level of consciousness. Repeat MRI after admission still showed no obvious abnormalities on DWI, T2-weighted imaging, susceptibility-weighted imaging (SWI), magnetic resonance angiography (MRA), or enhanced T1 imaging; however, arterial spin labeling (ASL) on the 14th day revealed markedly increased blood flow to the left hemisphere (Figure 2).

After a clinical course of recurrent seizures, transient fever in the 38°C range gradually occurred, with LDH (739 U/L) and sIL-2R (11,738 U/mL) elevating progressively during the next three weeks (Figure 1A). Contrast-enhanced computed tomography (CT) scans were negative for the presence of IVLBCL outside the cranium. Random skin biopsy and bone marrow examination revealed CD20-positive large atypical cells, confirming the diagnosis of IVLBCL. On the 27th day of admission, cyclophosphamide, hydroxydaunorubicin, vincristine, and prednisolone (CHOP) therapy was initiated; however, the patient developed tumor lysis syndrome. Subsequently, multiple organ failure progressed, and the patient died on the 43rd day of admission (Figure 1B).

## Discussion

We reported a rare case of IVLBCL with refractory seizures as the initial presenting symptom, which was also unique in that the patient showed the characteristic findings of unilateral hemispheric hyperperfusion on ASL as the only abnormal finding observed on MRI. EEG did not meet the Salzburg Consensus Criteria for Non-Convulsive Status Epilepticus (NCSE); however, repeated transient neurological deficits without lucid interval were consistent with a refractory seizure.

It is well known that IVLBCL often affects the CNS and causes the growth of large cells within the lumen of all sizes of vessels, leading to image findings such as ischemic stroke, tumors, or microbleeds detected on MRI [3-5]. However, our case did not show these typical findings. Instead, we observed notable hyperperfusion in the hemisphere on ASL, and no similar case, to our knowledge, has been reported so far.

ASL is a noninvasive MRI technique that semiquantifies cerebral blood flow by magnetically labeling arterial blood water as an endogenous tracer. It is recognized for its utility in diagnosing various pathological conditions, including cerebrovascular diseases, tumors, seizures, and inflammatory diseases. However, evidence of ASL findings in patients with IVL is scarce. Several mechanisms can be considered for the unilateral hemispheric hyperperfusion on ASL as the sole abnormal finding on MRI in this case. One possibility is that cerebral blood flow increased secondary to seizures, which could also be associated with heightened metabolic demand from tumor cells localized in the cortex. Although we could not confirm this through autopsy, it suggests that there might be a distinct pattern of CNS involvement in IVLBCL and that ASL may be beneficial in diagnosing this rare type of IVLBCL [6].

The findings of hyperperfusion in ASL can also occur in the pathologies of encephalitis and tumors [7]. Due to deterioration in the overall condition, we could not perform follow-up ASL imaging; but considering the symptom fluctuations observed, we thought the seizures to have had the greatest impact. Additionally, the observed hyperperfusion throughout the unilateral hemisphere suggested focal seizures.

## Conclusions

We reported a rare case of IVLBCL presenting with seizures as the initial symptom and unilateral hemispheric hyperperfusion in ASL as the only abnormal finding on brain MRI. This case highlights the need to consider IVLBCL when the patient's first seizure becomes a difficult-to-treat condition, even in the absence of its typical clinical findings. In such cases, clinicians should pay particular attention to the laboratory trends including LDH and sIL-2R for the early diagnosis and intervention.
